# Reduced Graphene Oxide/Polymer Monolithic Materials for Selective CO_2_ Capture

**DOI:** 10.3390/polym12040936

**Published:** 2020-04-17

**Authors:** Nikolaos Politakos, Iranzu Barbarin, Tomás Cordero-Lanzac, Alba Gonzalez, Ronen Zangi, Radmila Tomovska

**Affiliations:** 1 POLYMAT and Departamento de Química Aplicada, Facultad de Ciencias Químicas, University of the Basque Country UPV/EHU, Joxe Mari Korta Center—Avda. Tolosa, 72, 20018 San Sebastian, Spain; iranzu.barbarin@ehu.eus; 2 Department of Chemical Engineering, University of the Basque Country (UPV/EHU), P.O. Box 644, 48080 Bilbao, Spain; tomas.cordero@ehu.es; 3 POLYMAT, Department of Polymer Science and Technology, Faculty of Chemistry, University of the Basque Country, P.O. Box 1072, 20080 Donostia-San Sebastián, Spain; alba.gonzalez@ehu.eus; 4 POLYMAT and Department of Organic Chemistry I, Facultad de Ciencias Químicas, University of the Basque Country UPV/EHU, Joxe Mari Korta Center—Avda. Tolosa, 72, 20018 San Sebastian, Spain; r.zangi@ikerbasque.org; 5 IKERBASQUE, Basque Foundation for Science, Maria Diaz de Haro 3, 48013 Bilbao, Spain

**Keywords:** reduced graphene oxide, polymer latex, functionalized polymer nanoparticles, carbon dioxide capture, monoliths, porous materials

## Abstract

Polymer composite materials with hierarchical porous structure have been advancing in many different application fields due to excellent physico-chemical properties. However, their synthesis continues to be a highly energy-demanding and environmentally unfriendly process. This work reports a unique water based synthesis of monolithic 3D reduced graphene oxide (rGO) composite structures reinforced with poly(methyl methacrylate) polymer nanoparticles functionalized with epoxy functional groups. The method is based on reduction-induced self-assembly process performed at mild conditions. The textural properties and the surface chemistry of the monoliths were varied by changing the reaction conditions and quantity of added polymer to the structure. Moreover, the incorporation of the polymer into the structures improves the solvent resistance of the composites due to the formation of crosslinks between the polymer and the rGO. The monolithic composites were evaluated for selective capture of CO_2_. A balance between the specific surface area and the level of functionalization was found to be critical for obtaining high CO_2_ capacity and CO_2_/N_2_ selectivity. The polymer quantity affects the textural properties, thus lowering its amount the specific surface area and the amount of functional groups are higher. This affects positively the capacity for CO_2_ capture, thus, the maximum achieved was in the range 3.56–3.85 mmol/g at 1 atm and 25 °C.

## 1. Introduction

Currently, one of the major issues humanity is facing is climate change and the ramifications it brings on the whole life on the planet [1,2]. Despite all the efforts to develop alternative energy solutions, the majority of the world energy is still supplied from fossil fuel power plants, which cover 85% of the world energy demands and are responsible for an emission of 40% of the CO_2_ released in the atmosphere [1]. CO_2_ is one of the major players in the climate change, contributing to 80% of global warming effects [3]. Taking into account that approximately 37 billion metric tons of annual anthropogenic CO_2_ emissions in 2017 far out weight its utilization by over a factor of 100 [4], CO_2_ capture and sequestration (CCS) continue playing a key role in reducing the atmospheric CO_2_ concentration. Therefore, the development of effective technologies to reduce atmospheric CO_2_ concentration is an urgent and important task to make the transition towards green energy more smoothly [4]. 

Several technologies already exist to separate CO_2_ from the flue gas streams: solvent absorption, physical adsorption, membrane separation, cryogenic fractionation and chemical looping [5,6,7]. Liquid absorption is the most widely used technology for applications in CCS, however, both high energy required for regeneration and corrosiveness of amine solution absorbents are the major drawbacks [8]. Lately, solid adsorbents have arisen as an alternative and various types of solids have been used for the aim of CO_2_ capture, such as silica [9], zeolites [10], MOFs [3,11], mesoporous material such as MgO [11], metal decorated phosphorene [12], nanostructured copolymer and ionic liquids [12]. These materials showed very good performance in CO_2_ capturing due to their high specific surface area and the uniformity and tunability of their internal pores, however due to lack of feasibility of the scale-up of the synthesis procedures and lack of stability in cycle operations, their application is still on the laboratory level. In addition, zeolites present low stability in the presence of water vapor, which is usually present in the flue gasses after combustions [13,14]. Inorganic and polymeric membranes are investigated for CO_2_ capture as well, nevertheless their preparation is complicated and expensive [15]. In addition, the performances of polymeric membranes are limited by the “upper bound” due to the inverse relationship between permeability and selectivity [15].

In comparison to other solid adsorbents, carbon-based materials such as nanotubes [16], graphene [17], carbon spheres [18], activated carbon and activated carbon fibers [19] are appropriate for CO_2_ capture because they are characterized by a high stability in cycle operation and work better at higher pressures. Among the used carbon compounds, graphene and graphene oxide (GO) have attracted attention due to their superior thermal and mechanical properties, chemical stability and huge surface area [11,20,21]. The ability of graphene and GO to form monolithic materials and significantly increase the amount of active material per projected area resulted in development of novel class of materials with increased interest for selective CO_2_ capture, due to hierarchical porous structure [22,23,24,25,26,27,28]. In order to decrease the carbon footprint during production of these monolithic materials, milder synthesis conditions are favorable, under which the mechanical properties of the monolithic structures decreased [29]. This may affect the durability of the monoliths in cycle operation. Indeed, addition of polymers to support the monolithic graphene structure improved the physico-chemical and mechanical properties [20,21,30]. In addition, graphene based materials are characterized by intermediate binding or adsorption strength and provide mostly physisorption of the CO_2_ molecules [14], therefore addition of polymers may be a route to alter the surface chemistry of the monolithic structure and their affinity toward CO_2_ molecules. The combination of GO with polymers can lead to a synergistic effect of benefits from both materials, like excellent mechanical flexibility, high electric conductivity, and large specific surface area from the part of graphene and with the specific functions of diverse polymers. Polymers are a class of materials, which in their own right, extensively investigated for CO_2_ capture [31,32]. It has been demonstrated that important properties of the polymer adsorbent that determine the strength of the interactions with CO_2_ are composition, porous morphology and basic character [31]. The presence of heteroatoms such as N, S or O within polymer chains can promote a selective capture [31,32]. That is why polymers with heteroatoms were used to reinforce the neat monolithic graphene structure, such as polyethylenimine (PEI) [20], polythiophene [33], polypyrrole [34,35], chitosan [36] or polyaniline [2]. Few different methods of constructing these monolithic porous composites have been reported. Polymers have been used as a cross-linker during the self-assembly of GO sheets [20], however this method is feasible only for polymers containing suitable functionalities able to establish covalent bonds with GO. The most often used approach is the mixing of monomers with GO, followed by monomer polymerization and GO reduction steps [2,33,34,35]. However, this strategy exhibits high possibilities of the presence of toxic monomer residues into the final structure, which requires a number of time-consuming and costly purification steps that decreases the feasibility of the scale-up processes. Direct mixing of the polymer with GO solutions to achieve uniform dispersion, followed by a reduction self-assembly of GO was used for the synthesis of these materials as well [20]. Nevertheless, this method utilizes solvents, which are volatile organic compounds, and ultimately, released to the atmosphere. Considering these disadvantages of the techniques developed for synthesis of monolithic graphene/polymer materials for CO_2_ capture, scale-up procedures are either impossible or not viable. Still, there is a need for the development of environmentally friendly and low-energy method for the synthesis of this class of composite materials suitable for CO_2_ capture.

In this work, we present a unique latex based technique for the synthesis of composite polymer/graphene monolithic materials for CO_2_ selective capture. According to this technique, polymer latex (aqueous colloidal dispersion of polymer nanoparticles in water) is used as a matrix for the self-assembly process of GO platelets. The self-assembly is induced by ascorbic acid reduction reaction of GO. In this process, along with the assembly of rGO platelets incorporation of the polymer nanoparticles within the final monolithic structure occurred. This technique is environmentally friendly as the synthesis is exclusively performed in water, low energy (the structures are formed in temperature range of 45–90 °C, by self-assembly) and quite versatile. The versatility is owed to the wide range of polymers that may be incorporated in the monolithic structures, including highly hydrophobic ones, for which otherwise use of solvents is inevitable. In addition, latex technology permits introduction of various functionalities onto the polymer particles in relatively easy way during polymer synthesis by emulsion polymerization.

However, theoretical study of CO_2_ capture in reduced graphene oxide (rGO)/polymer system has shown that the addition of functionalized polymers to rGO structure decreases the capacity of neat rGO to capture CO_2_, by covering its surface [37]. This was confirmed in preliminary experiments, in which poly(methyl methacrylate) (PMMA) was functionalized using 1 wt % functional monomers. However, the theoretical calculations showed that by increasing the level of functionalization of the polymers the CO_2_ capture shifted upwards.

Herein, polymer nanoparticles made of MMA polymer functionalized with epoxy functionalities were used, synthesized by batch emulsion polymerization of monomer mixture MMA and glycidyl methacrylate (GMA) in weight ratio 90/10. MMA polymer was elected due to its high glass transition temperature that allows keeping the spherical particle nature over the rGO nanoplatelets after drying of the structures. This minimizes the coverage of the rGO surface. Epoxy functionalities were selected in order to take advantage of their CO_2_-filicity [38]. Therefore, synergistic action may be expected from functionalized graphene (rGO) and functionalized polymer. To design monoliths with high CO_2_ capture capacity and high selectivity, the reaction conditions and quantity ratios of the components in the composite monoliths were varied. The resulting monoliths presented hierarchical porous morphology with abundant amount of oxygen functionalities on the surface that allow selective capture of the CO_2_, and excellent stability during four cycles of adsorption/desorption. The environmental friendly nature of the synthesis method and mild conditions, along with excellent materials characteristics and potential for selective capture of CO_2_, make scale-up process highly viable.

## 2. Materials and Methods 

### 2.1. Materials

In this study graphene oxide (GO) was purchased from Graphenea, San Sebastian, Spain, as GO aqueous dispersion with concentration of 4 mg/mL, with monolayer content >95%, and a pH in a range between 2.2 and 2.5. The elemental analysis of GO, showed C: 49–56%, H: 0–1%, N: 0–1%, S: 2–4% and O: 41–50%. For the reduction of GO, L-Ascorbic acid (AsA, ≥99.0%, Sigma-Aldrich, Madrid, Spain) was used. Technical grade methyl methacrylate (MMA, Quimidroga, Barcelona, Spain) and glycidyl methacrylate (GMA; ≥97.0%, Sigma-Aldrich, Madrid, Spain), were used without further purification for synthesis of epoxy functionalized polymer nanoparticles. Potassium persulfate (KPS) as water soluble radical initiator, sodium dodecyl sulphate (SDS) as anionic surfactant and sodium bicarbonate (NaHCO_3_) buffer were supplied by Aldrich, Madrid, Spain and used as received. Deionized water was used as polymerization media.

### 2.2. Polymer Latex Synthesis

Epoxy functionalized PMMA latex was synthesized by batch emulsion polymerization of monomer mixture made of MMA/GMA in ratio 90/10 wt %. The formulation for a synthesis of 20% solid content latex is presented in Table 1.

The synthesis was performed in a 250 mL reactor equipped with a stainless steel impeller, a reflux condenser, and a N_2_ inlet. The monomer mixture was added in aqueous solution of SDS and stirred for 15 min at room temperature (23 °C) to prepare a course emulsion. Then the temperature of the reactor was raised to 70 °C and the aqueous solution of KPS/NaHCO_3_ were added as a shot and left to polymerize under inert atmosphere of N_2_ for 1.5 h. As a result, a latex with average polymer particle diameter of 70 nm was obtained with monomer conversion of 99%.

### 2.3. Preparation of Composite Porous Monolithic rGO/Polymer Materials

**Scheme 1:**
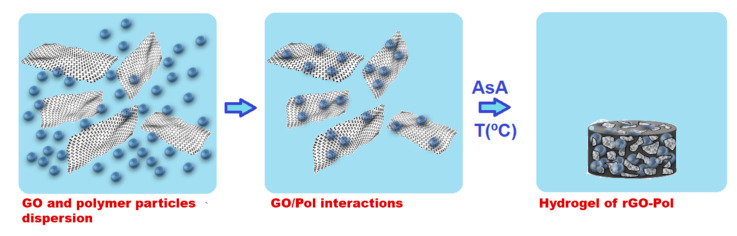
Schematic representation of the preparation of the monolithic sponge-like rGO/polymer composite.

Schematically the synthesis procedure is presented in Scheme 1. GO aqueous dispersion was sonicated using Hielscher Sonicator-UIS250v (amplitude of 70% and energy pulsed at 0.5 Hz, Hielscher Ultrasonics GmbH, Teltow, Germany) at 25 °C for 1 h. Then, the dispersion was transferred into a flask and left stirring for 2.5 h at 80 °C. Appropriate amount of this pre-treated GO aqueous dispersion and polymer nanoparticles dispersion (latex) were mixed for 3 h to allow adsorption of polymer nanoparticles over the GO nanoplatelets. Subsequently, the reducing agent AsA was added to the mixture and agitated mildly for 0.5 h. The reduction reaction was performed in an oven at three different temperatures 45 °C for 24 h, 60 °C for 4 h and 90 °C for 2 h. Two different GO:AsA weight ratios were used (a) 1:1 and (b) 1:2, and two different amounts of polymer with respect to GO were added: (1) 10 wt % and (2) 40 wt %. The materials produced and conditions of their production are listed in Table 2. After the reduction, 3D monolithic structures swelled with water were formed, as shown in Scheme 1. 

The formed wet structures were purified by dialysis process, the progress of which was followed by measuring water conductivity. The dialysis process was finished when conductivity values lower than 10 µS/cm was obtained. The wet monoliths were dried by freeze-drying technique using a Telstar LyoQuest 55 (Terrassa, Spain) at −49 °C and 0.2 mbar for 3 days. For comparison, the neat polymer latex was as well dried by freeze-drying technique, resulting in powdered samples, named “neat polymer”.

### 2.4. Characterization

The surface morphology of the monolithic composites was examined using a scanning electron microscopy (SEM,): a Hitachi TM3030 tabletop model (Krefeld, Germany) at an accelerating voltage of 15 kV after the samples were coated with a gold thin layer.

The porous texture of the monoliths was characterized by means of N_2_ adsorption–desorption at –196 °C in a Micromeritics ASAP2010 apparatus (Madrid, Spain). Prior to the analysis, the materials were degassed at 150 °C during 8 h under vacuum. From N_2_ adsorption–desorption isotherms, the specific surface area (S_BET_) was calculated from the Brunauer–Emmett–Teller (BET) equation. Moreover, *t*-plot method was used for estimating the micropore volume (V_micr_) and the external surface (S_ext_). The mesopore volume (V_mes_) was computed by difference between the total pore volume (V_T_) and micropore volume. Finally, the pore size distribution (PSD) and average pore diameter (d_p_) were calculated using the method proposed by Barrett–Joyner–Halenda (BJH method).

To determine the chemical composition and possible incorporation of polymer structure within the composite monolithic materials, solid-state ^13^C NMR spectra were recorded by a Bruker 400 AVANCE III WB spectrometer (9.40 T, Madrid, Spain) for 64 h at the resonance frequency of 100.6 MHz, using standard Bruker double-resonance magic angle sample spinning (MAS) probe. The samples were packed into a cylindrical zirconia rotor (4 mm external diameter), and then they were spun at a MAS frequency of 10 kHz. Spectra of solid samples were recorded using the high power decoupled (HPDEC) ^13^C pulse sequence, a time domain of 2 K, a spectral width of 55 kHz and an interpulse delay of 5 s.

Thermogravimetric analyses were performed in a TGA500 apparatus (TA Instruments, Cerdanyola del Valles, Spain) in order to estimate the amount of residual oxygen groups within the monolithic structure and to study the thermal stability of the monolithic composites. Samples of approximately 2 mg were heated under air atmosphere (90 mL/min) from 25 to 700 °C, at a rate of 10 °C/min. 

Solvent resistance of the monoliths was analyzed using tetrahydrofuran (THF). Samples of M90-2-10 and M90-2-40 monoliths were placed in THF and kept 24 h at 60 °C under agitation. The behavior of the composite monoliths was compared with the neat reduced graphene oxide (rGO) structure obtained under the same conditions.

For the CO_2_ adsorption experiments, a Cahn digital recording microbalance D-200 from Cahn Instruments, Inc. (Barcelona, Spain) with a resolution of 0.1 µg was used. The experiment was conducted in a thermally controlled environment with a temperature regulator (Conatec 4400), and a Vacuum Gauge Controller (Leybold Inficon Cm3 850-500-g1, precision of 1 mm Hg, Leybold Inficon, Bad Ragaz, Switzerland) connected with a vacuum pump (Varian DS102, Varinan, Palo Alto, CA, USA). Initially, the materials were weighted until a stable value of the mass was obtained. Subsequently, they were kept under vacuum overnight (15 h approximately) and weighted again to obtain the value of the initial mass. Then, the materials were flushed with CO_2_ (99.995% purity) until a pressure of 1 atm and temperature of 25 °C.

To determine the selectivity of the monolithic composites to absorb CO_2_ over that of N_2_ and to check the stability of the monoliths under cycle operation a TGA/DSC 3+ (Perkin Elmer, Waltham, MA USA) was used. Concerning the mass resolution of the equipment, this was 0.001 mg with a precision of 0.0025% and an accuracy of 0.005%. The temperature resolution was 0.001 K with a precision of ±0.2 K and accuracy of ±0.3 K. Prior to the measurements, the materials were placed in N_2_ atmosphere at 100 °C for 30 min. Afterwards, CO_2_ and N_2_ adsorption measurements were performed at 25 and 60 °C at atmospheric pressure and at a flow rate of the gasses of 50 mLmin. To assess the stability of the monoliths five adsorption/desorption cycles were carried out. Between each cycle, the monoliths were exposed to pure N_2_ flow (50 mL/min) for 30 min at 100 °C, in which period the initial mass of the samples was always recovered.

## 3. Results and Discussion

The self-assembly process of GO platelets occurring within the polymer latex is a complex hierarchical process, where the interactions between two different types of materials (polymer nanoparticles and GO) result in the formation of porous sponge-like monolithic composite materials. The composition of the monoliths was evaluated by ^13^C NMR and a representative spectra of the neat rGO and the composite structures are shown in Figure S1, Supporting information, demonstrating incorporation of both rGO and polymer within the composite structure. The porous properties of the monolithic materials depend on the process conditions and the ratios between the quantities of the starting materials. The resulting structures present physical consistency and monolithic appearance. The interplay between the good porous structure and suitable surface chemistry seems to be a tool towards enhancing the CO_2_ capture capacity. Therefore, the primary objective is to determine the morphology, porous structure (size and pore size distribution) and composition of each sample and to relate them to the capacity for the CO_2_ capture in a kind of experiential structure–performance relationship.

**Figure 1:**
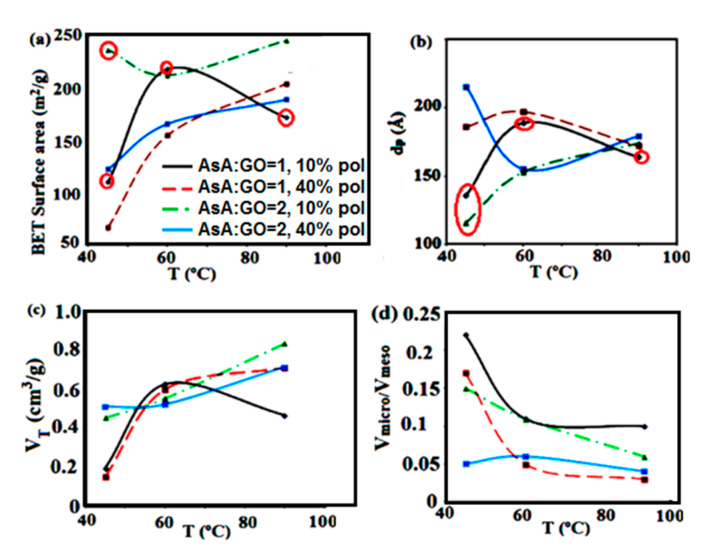
Dependence of the (**a**) Brunauer–Emmett–Teller (BET) surface area; (**b**) average pore diameter; (**c**) total pore volume and (**d**) volume ratio of micro- to mesopores of the monolithic composite materials on the reduction temperature at different amounts of AsA and polymer. Marked in red: the materials that present the best CO_2_ capture performance.

The textural properties of the monolithic materials were determined from N_2_ adsorption–desorption behavior (Figure S2), which provided detailed information on specific surface area, and textural parameters presented in Figure 1, Table S1, and Figure S3. The adsorption–desorption isotherms (Figure S2) are of type IV, typical of mesoporous materials. The shape of the isotherms depends on the synthesis conditions (temperature, AsA and polymer quantities) and reveals details on the morphology of the monolithic materials. At low reduction temperatures (45 °C, Figure S2a) or lower amount of AsA at 60 °C (Figure S2b), the isotherms exhibited H2 hysteresis loops, indicating cylindrical pores with narrow mouths morphologies. Nevertheless, pores were lamellar and V-shaped (H3 hysteresis loop) for samples prepared at 90 °C (Figure S2d) with higher AsA content at 60 °C (Figure S2c). 

Figure 1 reveals the dependence of the textural parameters of the materials on reduction temperature, different amounts of AsA and polymer. When the reduction was performed at 45 °C, the AsA and polymer amount presented an important effect on the structural parameters, therefore the BET surface area changed in the range of 67–235 m^2^/g (Figure 1a). By increasing the temperature this effect was lower, therefore at 90 °C the BET surface areas were changed in a small range of 171–244 m^2^/g. As the reduction reaction was very fast at higher temperatures, it seemed that the textural characteristic of these materials were formed in the first phase of the self-assembly process, independently on the quantity of AsA or the polymer. 

**Figure 2:**
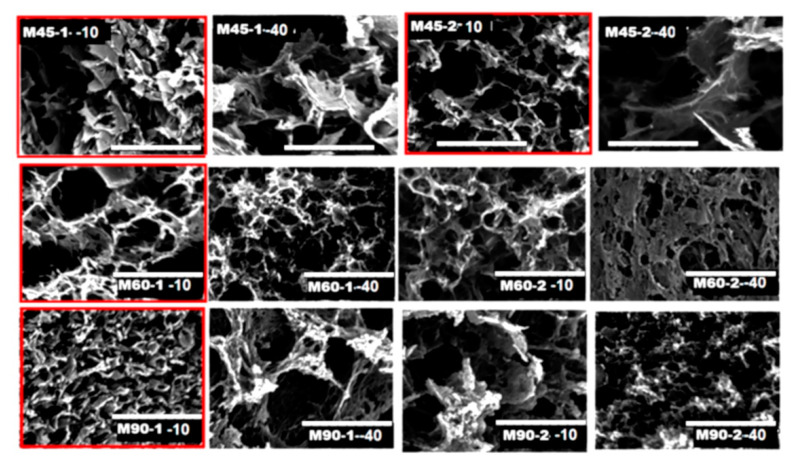
SEM images of composite monoliths. The scale bar in each image is 30 mm. The structures with maximum CO_2_ adsorption are marked in red.

The SEM images of the material produced at 45 °C (Figure 2) present larger pores than the structures produced at 60 °C (Figure 2, top) This type of porosity could allow easier adsorption of CO_2_ in the initial stages of the process. Figure 1c, where the total volume of the pores is shown as a function of temperature, indicates that the materials produced at 45 °C were less porous, however, they had a larger fraction of mesopores (see micro- to mesopores volume ratios in Figure 1d). As the temperature of the reduction process increased, the materials had more compact morphology, the total pore volume and porosity was increased, but the fraction of mesopores dropped (Figure 1c). 

The amount of AsA used for reduction is an important parameter that influences the porous properties, especially at lower temperatures, when this amount determines the reduction velocity and subsequently the self-assembly of the platelets. In general, lower amount of AsA (AsA:GO = 1, red and black curves in Figure 1, respectively) gives rise to higher BETs, lower average pore diameter and higher fraction of mesopores. The amount of polymer also affects the self-assembly process, as the polymer nanoparticles in dispersions are adsorbed at the GO platelets prior to reduction (Scheme 1). This affects the mobility and the affinity to self-assembly of the nanoplatelets, which form structures that are more robust. In general, an increase in the amount of polymer is expected to decrease the reduction rate, obtaining less compact structures even at higher temperatures and AsA amounts (as it is the case with M60-1-40, M60-2-40 or M90-2-40. These monoliths exhibit a decrease in the fraction of mesopores, and materials with lower porosity are produced (Figure 1c,d). According to the results in Figure 1d an increase in the polymer amount leads to the microporosity detriment and mesoporosity increase, without significant changes of the total pore volume (Figure 1c). 

Apart from the information of thermal stability, an estimation of chemical composition was obtained from the thermal degradation curves of the monolithic materials presented in Figure S4. According to these results, the materials synthesized at higher temperature present higher thermal stability due to less of an amount of organic moieties presented within the porous structure. This effect was more pronounced at lower amounts of AsA and polymer (materials with AsA:GO = 1 and 10% of polymer in Figure S4). The thermal stability at lower temperatures (<200 °C) was enhanced for a higher amount of polymer, however, it dropped significantly at higher temperatures (>200 °C). This is an important property of these monoliths for their practical application, if we take into account that the temperature rang in which these monoliths will be practically applied is 50–160 °C. Table 3 lists the weight loss distributed by temperature ranges. By comparison of neat rGO and neat polymer TGA (not presented here) we developed a strategy to determine the chemical composition of the rGO monoliths. The weight loss at temperatures lower than 225 °C was attributed to the oxygen functional groups from rGO and the weight loss between 225 and 425 °C corresponds to the polymer present within the structures. Under temperature higher than 465 °C, the weight loss was attributed to the basal carbon skeleton. From Table 3 it is clear that this amount was much higher when 10% polymer was added to the composites than in case of 40% polymer. 

**Scheme 2:**
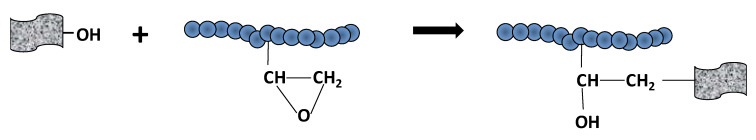
Addition reaction polymer-GO through OH-epoxy reaction (OH may originate from COOH, too).

According to Table 3, the content of functional groups within the materials produced at lower reduction temperatures (series of 45 °C and 60 °C) was high. The same effect was observed for lower amount of reducing agent AsA (AsA:GO = 1). Surprisingly, the amount of functionalities left onto rGO after reductions was significantly affected by the polymer quantity (comparing materials with 10% and 40% polymer). In materials containing higher polymer amount, the quantity of residual oxygen functional groups onto rGO was lower. This effect indicates that there were certain interactions between polymer and oxygen functional groups that caused their waste. We hypothesized that covalent bonding occurred between GO and polymer chains. A possible route may be the presence of epoxy functionalities onto polymer nanoparticles that react with -OH or -COOH from GO under acidic pH and high temperature, according to Scheme 2.

**Figure 3:**
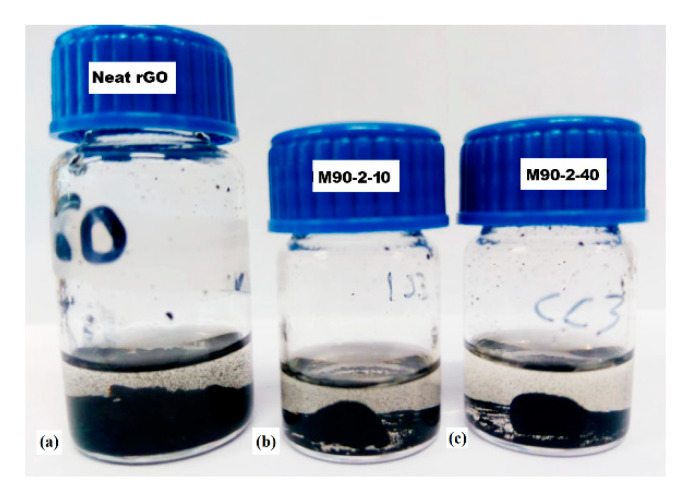
Solubility test in THF of (**a**) neat rGO monolithic structure: (**b**) M90-2-10 and (**c**) M90-2-40.

Evidence of polymer-rGO covalent bonding has been reported previously in similar systems [39,40]. In order to check this hypothesis, solubility tests were performed using tetrahydrofuran (THF) solvent, selected because it is a good solvent for the MMA/GMA linear chains. The neat monolithic rGO structure was expected to collapse within THF. Figure 3 shows photos of these materials after the solubility test. Figure 3a indicates that the neat graphene monolithic material indeed collapsed and the powdered sample precipitated in THF. In Figure 3b,c one may observe that a part of both M90-2-10 and M90-2-40 decomposed into powdered sample. Nevertheless, consistent monolithic pieces may be observed in both cases, even after the temperature treatment of the THF/monoliths mixture at 60 °C for 24 h. The ability to keep the monolithic appearance within THF even at prolong period of time at high temperature of the composite monoliths is likely due to partially cross-linked monolithic arrangements.

The weight loss between 265 and 425 °C is attributed to the polymer within the structures. Nevertheless, the weight loss in this temperature range (16–62%) was higher than the polymer content in the materials, which is either 10% (M45-1-10, M45-2-10, M60-1-10, M60-2-10, M90-1-10 and M90-2-10) or 40% (M45-1-40, M45-2-40, M60-1-40, M60-2-40, M90-1-40 and M90-2-40). This indicates that, along with the organic part a great part of rGO structure was decomposed. This loss was significantly postponed in the structures obtained at higher temperature and AsA quantity, as observed in Figure S4. These results are in accordance to the hypothesis of stable bonding between the polymer and rGO. The final residuals after TGA analysis at 700 °C decreased with synthesis temperature, which could confirm that samples synthesized at milder conditions were not so compact and their oxygen was consumed earlier during the analysis, producing larger residual amounts at higher temperatures.

**Figure 4:**
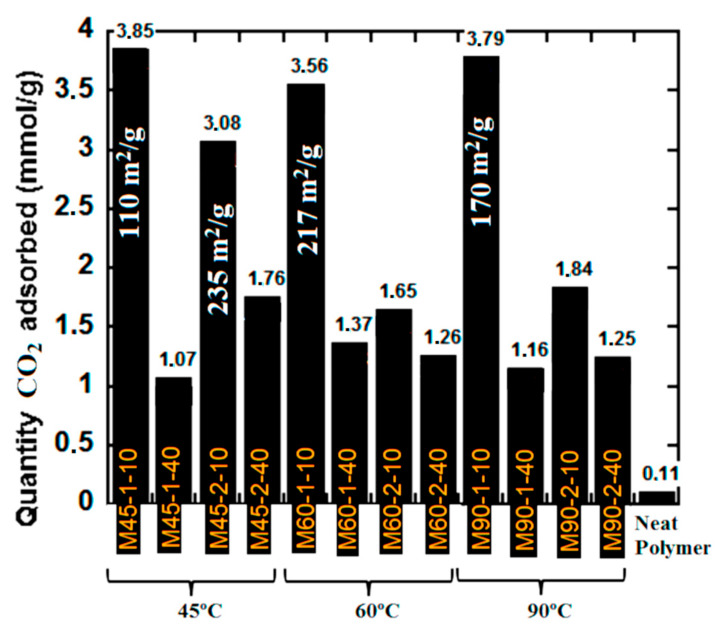
CO_2_ capture capacity of the monolithic composites at standard atmospheric conditions (T = 25 °C and P = 1 atm).

The CO_2_ capture performance of the monolithic composites is presented in Figure 4, where the data of CO_2_ equilibrium adsorption at atmospheric conditions (25 °C and 1 atm) are presented. 

According to Figure 4, the highest CO_2_ capture capacity were achieved by the following monoliths M45-1-10, M60-1-10 and M90-1-10, all of them synthesized at the lowest AsA and polymer quantity at all investigated temperatures. Under such conditions, the monoliths were richer with functionalities (Table 3) that likely promoted the interactions with CO_2_. The specific surface area of each of these materials is presented in Figure 4, ranging from 110 to 217 m^2^/g, indicating that the surface area was not the only determining factor for high performance. On the other hand, a relatively high performance for CO_2_ sorption was observed for M45-2-10 (3.08 mmol/g), which was produced at higher AsA amount and low temperature of reduction (45 °C). It presents one of the highest specific surface area obtained in this work (235 m^2^/g) and relatively high content of oxygen groups (5.5%), indicating that interplay between these two characteristics was crucial for the CO_2_-filicity. Another important parameter was the V_micro_/V_meso_ ratio that for all mentioned monoliths with high capacity to capture CO_2_ was high (Table S1, Supporting information).

Since the fluent gasses for post-combustion CO_2_ capture, beside CO_2_ were abundant in N_2_, the ability of the potential sorbents for selective CO_2_ adsorption over that of N_2_ was an indispensable characteristic. The monoliths with the highest CO_2_ capture capacity, M45-1-10, M60-1-10 and M90-1-10 were used for individual adsorption of CO_2_ and N_2_ selectivity measurements using TGA method. In order to study the selectivity at ambient conditions and a high temperature, as in the post-combustion fluent gas, the evaluation was performed at 1 atm at two different temperatures, 25 °C and 60 °C. Thereby, the equilibrium value of CO_2_ and N_2_ adsorption were obtained, from which the selectivity was calculated as a ratio of capture capacities for both gasses CO_2_ and N_2_. The values for both temperatures are presented in Table 4.

At 25 °C, M45-1-10 presents lower selectivity than M60-1-10 and M90-1-10, its higher content of functional groups (Table 2). Surprisingly, at 60 °C its selectivity increased, which is unusual and unexpected behavior that may be related to certain thermal instability of this monolith produced at a lower temperature (black curve in Figure S4a), which resulted in mass loss during the first measurement at 25 °C and an underestimated value of CO_2_ capture. According to Table 4, the monoliths synthesized at higher temperatures provide improved selectivity towards CO_2_ at 25 °C. At 60 °C the values of selectivity dropped, however not as drastically as reported in the literature [41]. Similar selectivity range was obtained computationally [37]. Moreover, all the monoliths have rather similar selectivity at 60 °C, probably due to a similar balance of the surface area and content of functionalities. The functional groups determine the interactions in general, however their distribution and density on the surface is determined by the available surface area. Namely, M45-1-10 had the lowest surface area of 110 m^2^/g and very high content of functionalities 19%, whereas M60-1-10 had a surface area of 217 m^2^/g and about 8% of functionalities. Finally, the surface area of M60-1-10 was 170 m^2^/g; however, the functionality content was 10%. Apparently, for such solid sorbents, this balance of surface area and surface functionalization is the controlling parameter for a good performance in CO_2_ capture. However, no trend was found between adsorption performance and the individual textural parameters (Figure 2 and Table S1), confirming that interplay between them and the functionalization is what determine the performance.

**Figure 5:**
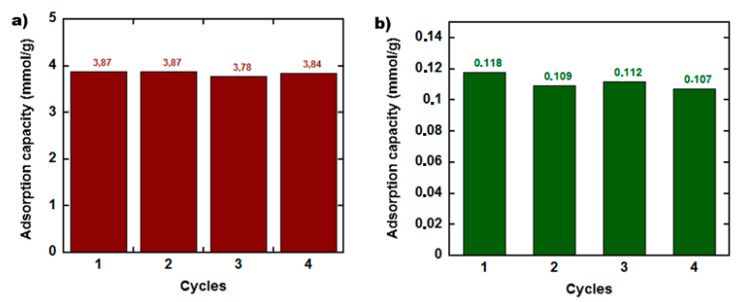
Four CO_2_ adsorption/desorption cycles at standard atmospheric conditions (T = 25 °C and P = 1 atm) of (**a**) M90-1-10 and the (**b**) neat polymer.

The advantage of using polymers within the composite monolithic structures is the enhanced robustness and consistency of the prepared materials, being simultaneously easy to handle. However, one of the major advantages of graphene-based materials for CO_2_ capture is the stability in cycle operation, the material regeneration and reuse, as the mechanism of CO_2_ capture is pure physical adsorption. On the other hand, polymer sorbents are characterized by combination of adsorption/absorption mechanisms, therefore regeneration may be challenging. To evaluate the capacity for regeneration of the monoliths, M90-1-10 monolith was selected to study the stability in cycle operations. For comparison, the neat polymer was studied as well. Four adsorption/desorption cycles were performed and the results are depicted in Figure 5a. After each cycle, M90-1-10 recuperated its original mass, without significant variation in the adsorption value of CO_2_ (the maximum lost in adsorption capacity was 0.7%). Figure 5b presents the adsorption/desorption behavior of the neat polymer, showing that the adsorption in last cycle decreased for about 9.3%, meaning that some quantity of CO_2_ was likely absorbed within the polymer and would need more energy than applied to desorb it. As the composite monolith contain 10% polymer, the desorption process was not affected and proceeded smoothly under pressure decrease. Furthermore, the capacity of the neat polymer to capture CO_2_ is about 40 times smaller than that of the composite material.

## 4. Conclusions

Monolithic porous composites, based on rGO/polymer, were prepared by self-assembly of rGO platelets within the matrix of polymer latex (aqueous dispersion of polymer nanoparticles). MMA polymer nanoparticles functionalized with epoxy functionalities at the surface were incorporated spontaneously during the self-assembly process and brought robustness, consistency and functionalization to the monolithic structures. By changing the reaction temperature and the amount of components, the textural properties, morphology and the level of functionalization were modulated. Such nanostructured monoliths were designed for selective CO_2_ capture.

The capacity for selective CO_2_ capture over that of N_2_ was evaluated and it was found that proper balance between the active specific surface area and the quantity of surface functionalities were crucial for high capacity and high selectivity. The maximum capacity for CO_2_ capture achieved was 3.85 mmol/g, whereas the selectivity determined as a simple ratio of capacity for CO_2_ and N_2_ was 12 at 25 °C and about 9 at 60 °C. Furthermore, the composite monoliths showed stability in cycle operation, demonstrated in four adsorption/desorption cycles, showing no mass loss (physical stability) and easy desorption of the complete amount of CO_2_ adsorbed. 

## Figures and Tables

**Table 1:** Formulation for synthesis of MMA/GMA 90/10 wt % polymer latex by batch emulsion polymerization.

MMA	GMA	H_2_O	SDS	NaHCO_3_	KPS + H_2_O
21.6 g	2.4 g	95 g	3% wbm ^a^	2.5% wbm	0.5% wbm + 6.8 g H_2_O

^a^ % wbm—% by weight based to monomer.

**Table 2:** Nomenclature of the monolithic materials and their synthesis conditions.

Samples	Temperature (°C)	GO:AsA (Mass Ratio)	Polymer Quantity (Mass %)
M45-1-10	45	1:1	10
M45-1-40	45	1:1	40
M45-2-10	45	1:2	10
M45-2-40	45	1:2	40
M60-1-10	60	1:1	10
M60-1-40	60	1:1	40
M60-2-10	60	1:2	10
M60-2-40	60	1:2	40
M90-1-10	90	1:1	10
M90-1-40	90	1:1	40
M90-2-10	90	1:2	10
M90-2-40	90	1:2	40

**Table 3:** Weight loss at specific temperatures: at 225 °C (oxygen functional groups), in the range of 265–425 °C (polymer degradation) and at 700 °C (final residue).

Samples	Weight Loss (%) <225 °C	Weight Loss (%) 265–425 °C	Weight Loss (%) 425–700 °C	Residuals (%) <700 °C
M45-1-10	19.5	31.3	46.6	+2.6
M45-1-40	8.3	62.6	28.5	+0.6
M45-2-10	5.5	34.4	51,7	+8.4
M45-2-40	7.3	58.7	25.8	+8.2
M60-1-10	7.8	41.4	50.1	+0.7
M60-1-40	8.3	62.5	28.6	+0.6
M60-2-10	4.2	16.2	73.4	+6.2
M60-2-40	7.1	58.7	25.9	+8.3
M90-1-10	10.1	28.0	61.0	+0.9
M90-1-40	3.4	60.8	34.8	+1.0
M90-2-10	3.2	45.0	51.0	+0.8
M90-2-40	3.3	57.7	38.4	+0.6

**Table 4:** The CO_2_/N_2_ selectivity of the three monoliths with the highest performance for CO_2_ capture.

Monolith	Selectivity (Capacity Ratio CO_2_ to N_2_)	
	25 °C	60 °C
M45-1-10	5.9	8.7
M60-1-10	11.6	7.5
M90-1-10	12.2	9.2

## Supplementary Materials

The following are available online at https://www.mdpi.com/2073-4360/12/4/936/s1, Figure S1: Solid-state ^13^C NMR for the neat rGO structure (left) and M60-1-40 (right) obtained during reduction at 60 °C; Figure S2. Absorption-desorption isotherms of various monolithic materials; Figure S3. Pore size distributions of the different monolithic materials; Figure S4. TGA curves of composite monolithic materials, obtained at: (a) 45 °C; (b) 60 °C; and (c) 90 °C. Table S1: Textural parameters calculated from N_2_ adsorption-desorption isotherms.
