# Supplementary material for: Reduced Graphene Oxide/Polymer Monolithic Materials for Selective CO2 Capture
Source: Polymers (Basel). 2020 Apr 17;12(4):936. doi: 10.3390/polym12040936 (PMC7240369; doi:10.3390/polym12040936)
Supplement: Supplementary file 1 [file polymers-12-00936-s001.pdf]

Supporting Information

Reduced Graphene Oxide/Polymer Monolithic Materials for Selective CO_2 Capture

Nikolaos Politakos [1,*], Iranzu Barbarin [1], Tomás Cordero-Lanzac [2], Alba Gonzalez [3], Ronen Zangi [4,5] and Radmila Tomovska [1,5,*]

1 POLYMAT and Departamento de Química Aplicada, Facultad de Ciencias Químicas, University of the Basque Country UPV/EHU, Joxe Mari Korta Center—Avda. Tolosa, 72, 20018 San Sebastian, Spain; iranzu.barbarin@ehu.eus (I.B.)

2 Department of Chemical Engineering, University of the Basque Country (UPV/EHU), PO Box 644, 48080 Bilbao, Spain; tomas.cordero@ehu.es (T.C.-L.)

3 POLYMAT, Department of Polymer Science and Technology, Faculty of Chemistry, University of the Basque Country, P.O. Box 1072, 20080 Donostia-San Sebastián, Spain; alba.gonzalez@ehu.eus (A.G)

4 POLYMAT and Department of Organic Chemistry I, Facultad de Ciencias Químicas, University of the Basque Country UPV/EHU, Joxe Mari Korta Center—Avda. Tolosa, 72, 20018 San Sebastian, Spain; r.zangi@ikerbasque.org (R.Z.)

5 IKERBASQUE, Basque Foundation for Science, Maria Diaz de Haro 3, 48013 Bilbao, Spain

* Correspondence: nikolaos.politakos@ehu.eus (N.P.); radmila.tomovska@ehu.es (R.T.)

Solid ^{13}C-NMR analysis

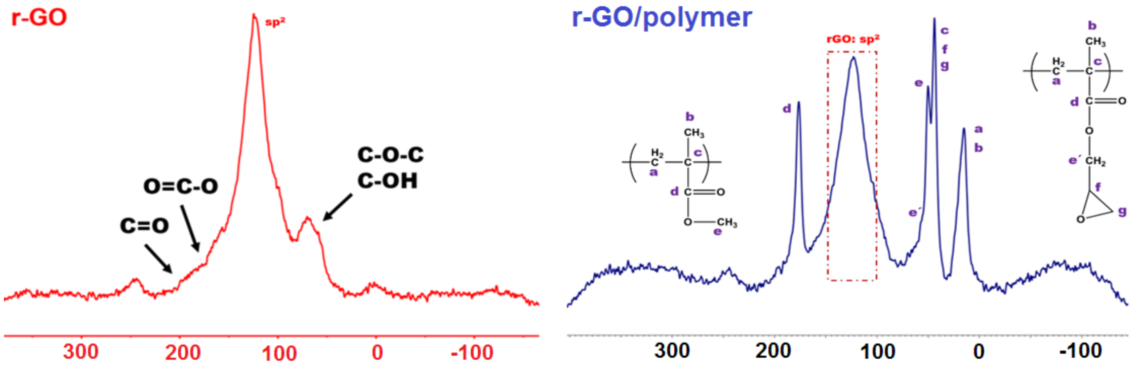

Figure S1. Solid-state ^{13}C NMR for the neat rGO structure (left) and M60-1-40 (right) obtained during reduction at 60ºC.

Adsorption-desorption of N2 results

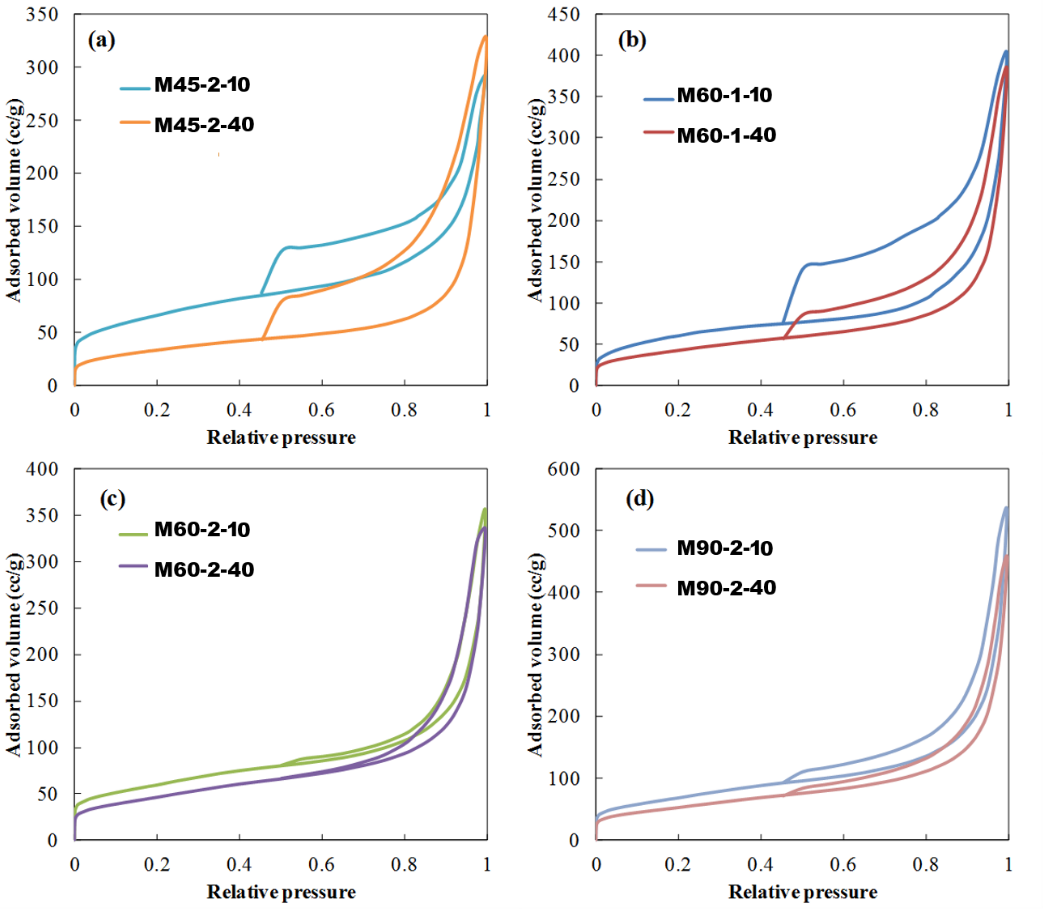

Figure S2. Absorption-desorption isotherms of various monolithic materials.

Table S1. Textural parameters calculated from N_2 adsorption-desorption isotherms.

Samples	S_{BET} (m²/g)	S_{ext} (m²/g)	V_{micr} (cm³/g)	V_{mes} (cm³/g)	V_{micr} / V_{mes} ratio	V_T (cm³/g)	d_p (Å)
**M45-1-10**	110	27	0.036	0.161	0.22	0.197	136
**M45-1-40**	67	18	0.022	0.129	0.17	0.151	186
**M45-2-10**	235	115	0.060	0.392	0.15	0.452	116
**M45-2-40**	122	66	0.026	0.482	0.05	0.508	215
**M60-1-10**	217	83	0.064	0.561	0.11	0.625	189
**M60-1-40**	154	102	0.026	0.570	0.05	0.596	197
**M60-2-10**	211	106	0.054	0.497	0.11	0.551	153
**M60-2-40**	165	114	0.028	0.492	0.06	0.520	155
**M90-1-10**	171	85	0.042	0.423	0.10	0.466	164
**M90-1-40**	203	140	0.034	0.973	0.03	0.707	172
**M90-2-10**	244	154	0.047	0.783	0.06	0.830	174
**M90-2-40**	188	140	0.025	0.685	0.04	0.710	179

From BJH method, the pore size distributions (PSD) of each sample were calculated and depicted in Figure S2. Figure S2a shows the PSD of samples with a GO:polymer analogy of 1:0.1 and Figure S2b displays those of samples with a GO:latex analogy of 1:0.4. Three contributions to the total

porosity are observed in different pore ranges for most of the samples. Nevertheless, the contribution of pores with diameters < 20 Å (micropore range) is negligible in comparison with those of meso- and macropore ranges. Two well-defined maxima are shown at ca. 475 (mesopore range) and ca. 1350 Å (macropore range). It is noteworthy the increase in macropore contribution upon increasing the preparation temperature, even being negligible the presence of macropores in several samples activated at 45 ºC (M45-1-10, M45-1-40 or M45-2-40)

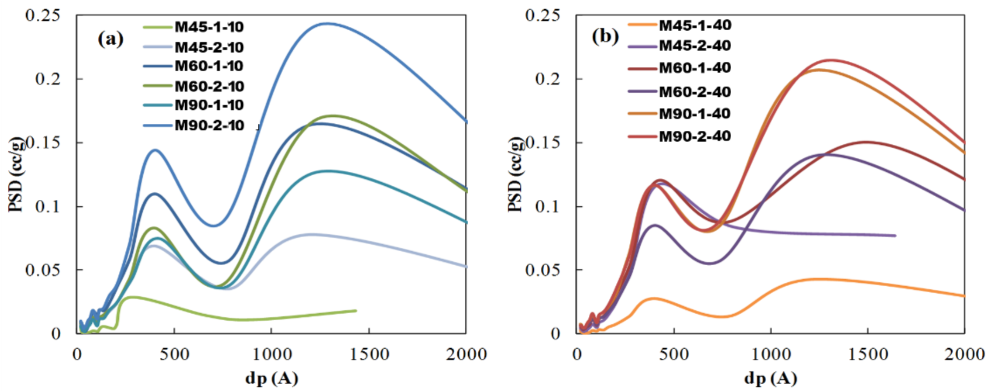

Figure S3. Pore size distributions of the different monolithic materials.

TGA analysis

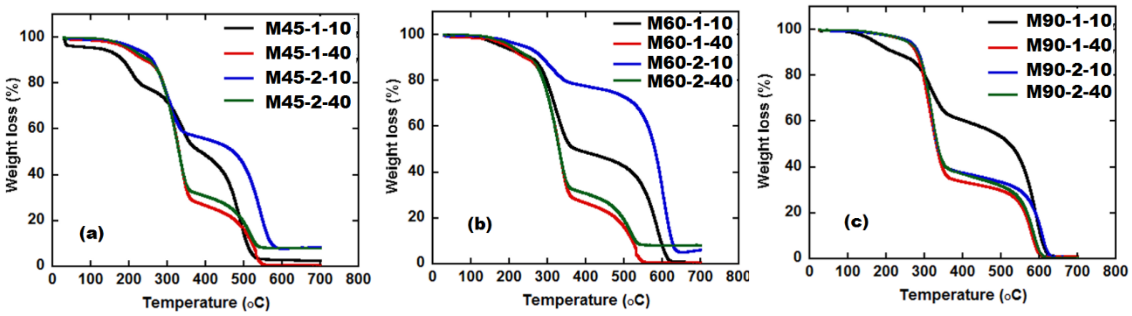

Figure S4. TGA curves of composite monolithic materials, obtained at: (a) 45ºC; (b) 60ºC; and (c) 90ºC.
